# Long-Term Prognostic Performance of Ki67 Rate in Early Stage, pT1-pT2, pN0, Invasive Breast Carcinoma

**DOI:** 10.1371/journal.pone.0055901

**Published:** 2013-03-19

**Authors:** Fabien Reyal, David Hajage, Alexia Savignoni, Jean-Guillaume Feron, Marc Andrew Bollet, Youlia Kirova, Alain Fourquet, Jean-Yves Pierga, Paul Cottu, Veronique Dieras, Virginie Fourchotte, Fatima Laki, Severine Alran, Bernard Asselain, Anne Vincent-Salomon, Brigitte Sigal-Zafrani, Xavier Sastre-Garau

**Affiliations:** 1 Department of Surgery, Institut Curie, Paris, France; 2 Department of Biostatistic, Institut Curie, Paris, France; 3 Départment of Radiation Oncology, Institut Curie, Paris, France; 4 Département of Medical Oncology, Institut Curie, Paris, France; 5 Département of Tumour Biology, Institut Curie, Paris, France; University of Porto, Portugal

## Abstract

**Background:**

Molecular signatures may become of use in clinical practice to assess the prognosis of breast cancers. However, although international consensus conferences sustain the use of these new markers in the near future, concerns remain about their degree of discordance and cost-effectiveness in different international settings. The present study aims to validate Ki67 as prognostic factor in a large cohort of early-stage (pT1–pT2, pN0) breast cancer patients.

**Methods:**

456 patients treated in 1995–1996 were identified in the Institut Curie database. Ki67 (MIB1) was retrospectively assessed by immunohistochemistry for all cases. The prognostic value of this index was compared to that of histological grade (HG), Estrogen receptor (ER) and *HER2* status. Distant disease free interval, loco-regional recurrence, time-lapse from first metastatic diagnosis to death were analyzed.

**Results:**

All 456 patients were treated by lumpectomy plus axillary dissection and radiotherapy. 27 patients (5.9%) received systemic treatment. Tumors were classified as HG1 in 35%, HG2 in 42% and HG3 in 23% of cases. ER was expressed in 86% of the tumors, *HER2* in 5% and 14% were triple negative. The median follow-up was 151 [5–191] months. Distant and loco-regional disease recurrences were observed in 16% and 18%, respectively. High (>20%) Ki67 rate [HR = 3 (1.8–4.8), p<10e−06] and HG3 [HR = 4.4 (2.2–8.6), p = 0.00002] were associated with an increased rate of distant relapse. In multivariate analysis, the Ki67 remained the only significant prognostic factor in the subgroups of ER positive *HER2* negative [HR = 2.6 (1.5–4.6), p = 0.0006] and ER positive HER2 negative HG2 tumors [HR = 2.2 (1.01–4.8), p = 0.04].

**Conclusions:**

We validate the prognosis value of the Ki67 rate in small size node negative breast cancer. We conclude that Ki67 is a potential cost-effective decision marker for adjuvant therapy in early-stage HG2, pT1–pT2, pN0, breast cancers.

## Introduction

Breast cancer prognostic factors are essential to identify patients at risk of distant metastasis development and to decide whether adjuvant treatments are needed. The most validated biological marker in non-metastatic breast cancer are tumor size, histological grade, mitotic index, Ki67 rate, axillary lymph node involvement, Estrogen Receptor (ER), Progesterone Receptor (PR) and *HER2* status. Tumor proliferation is one of the major factors associated with prognosis [11]. It can be measured by two widely used markers mitotic index (MI) and Ki67 rate. MI, defined as the number of mitoses per 10 high power fields at the periphery of the tumor [16], [17], carries the main part of the prognostic value of the histological grade (Nottingham system). This index is linked to the percentage of tumor cells undergoing mitosis and to the duration of the cell-cycle, considering that the M phase is only a short part of the cell-cycle process. However MI does not reflect the doubling time of the tumor. In a large meta-analysis of 20 studies [45] corresponding to 7,021 patients, the independent prognostic value of MI for metastases or cancer specific deaths in breast cancer patients was confirmed using univariate and multivariate models. Nuclear Ki67 immuno-staining is the second proliferation marker most widely used in clinical practice. The Ki67 protein is present during G1, S, G2, M phases of the cell cycle and is strictly associated with cell proliferation. The Ki67 rate is most often measured on histological sections and is defined as the percentage of stained invasive carcinoma cells. The prognostic value of the Ki67 rate has been confirmed in several meta-analyses including univariate and multivariate models [12], [13], [45], [53]. The Saint-Gallen guidelines [21], National Institute of Health guidelines [15] and Nottingham Prognosis Index guidelines [5] as well as the AdjuvantOnline! decision making tool [39] use a combination of these prognostic factors to assess the need for adjuvant treatments based. Owing to insufficiently accurate prognosis predictions, a substantial proportion of patients with breast cancer receive useless adjuvant systemic therapy [14]. High-throughput technologies such as gene expression microarrays offer new opportunities to improve the ability to determine prognosis for individual patients. Molecular signatures (Proliferation signatures) such as Mammaprint© (Agendia, Amsterdam, Netherlands) [46], [47], OncotypeDX© (Genomic Health, Redwood, California, USA) [35] and MapquantDX© (Ipsogen, Marseille, France) [44] may become of use in clinical practice. International consensus conferences seem to sustain the use of these new markers in the near future despite great concerns about the real benefit, the large degree of discordance between them and the potential low cost-effectiveness of these classifiers. In a pilot study, and taking into account medico-economic aspects, we favored the use of Ki67, with a 20% cut-off, as a routine marker for the assessment of tumor cell proliferation (Reyal et al., Plos one 2012). The present study aims at analyzing the Ki67 prognostic value in a large cohort of early-stage, pN0, breast cancer patients treated in a reference comprehensive cancer center. We focused our analysis on the ER positive *HER2* negative subgroup and on the ER positive *HER2* negative Grade 2 subgroup as they represent two entities with a need to improve their prognostic determination and consequently their adjuvant treatment decision-making process. Conversely, the treatments of patients with *HER2* positive or triple-negative tumour do not rely on the level of their proliferation markers due to the intrinsic aggressiveness of these two subgroups.

## Materials and Methods

Our dataset consisted of 456 early-stage (pN0) breast cancer patients treated between 1995 and 1996 by breast conserving surgery with axillary lymph node dissection as primary treatment at the Institut Curie and identified through the Institut Curie prospective breast cancer database. The main inclusion criterion was the absence of pathologic axillary lymph node involvement. Patients who had received a neoadjuvant treatment (chemotherapy, hormonal therapy or radiotherapy) were excluded from the study.

The histological features (Histological Type, Elston Ellis Grade, Mitotic Index, Ki67 rate, Estrogen Receptor status, Progesterone Receptor status, *HER2* over expression status) were re-assessed for each sample by senior pathologists. Tissue sections of 4 µm were prepared from a representative part of each tumor sample to score several markers.

### Mitotic Index

Mitotic Index was assessed on histological sections stained by Hematein, Eosin and Saffron. The criteria of Van Diest and al were used to define mitotic figures [48], [49]. It corresponded to the mitotic score defined in the Nottingham grade; the number of mitoses observed in 10 consecutive high power fields (HPF) using a microscope with 40× objectives and a 10× ocular. Cut-off, according to the field of our microscope, <10, 10–19 and ≥20 mitosis were used to define low, intermediate and high mitotic indexes.

### Ki67 rate

Tissue sections were first digested in 0.1% trypsin and 0.1% calcium chloride in triphosphate buffer saline pH 7.6 for 5 minutes. Antigen retrieval was performed by incubating tissue sections for 20 minutes in citrate buffer 10 mM (ph 6.1) in a 850 W microwave oven. Tissue sections were then incubated for one hour with the anti-Ki67 monoclonal antibody (Clone MIB1, Dako A/S, Glostrup, Denmark) at 1/100 dilution. The revelation of the staining was performed using the Vectastain Elite ABC peroxydase mouse IgG kit (Vector Burlingame, CA, USA) and diamino-benzidine (Dako A/S) as chromogen. The semiquantitative assessment was performed by estimating at X200 magnification, the percentage of positive neoplastic nuclei within the area of highest positivity chosen after scanning the entire tumour surface at low power (x10 objective). All nuclei with homogeneous staining even with a light staining or only a nucleolar staining were interpreted as positive. A cut-off of >20% was used to define tumors with high KI67 rate.

### Estrogen receptor (ER) and Progesterone receptor (PR) status

After rehydration and antigenic retrieval in citrate buffer (10 mM, pH 6.1), the tissue sections were stained for estrogen receptor (ER, clone 6F11, Novocastra, 1/200), and progesterone receptor (PR, clone 1A6, Novocastra, 1/200). Revelation of staining was performed using the Vectastain Elite ABC peroxidase mouse IgG kit (Vector Burlingame, CA) and diaminobenzidine (Dako A/S, Glostrup, Denmark) as chromogen. Positive and negative controls were included in each slide run. Cases were considered positive for ER and PR according to standardized guidelines using a cut-off of ≥10% stained tumour nuclei [3], [4].

### HER2 status

After rehydration and antigenic retrieval in citrate buffer (10 mM, pH 6.1), the tissue sections were stained for HER-2 (clone CB11, Novocastra, 1/1000). Revelation of staining was performed using the Vectastain Elite ABC peroxidase mouse IgG kit (Vector Burlingame, CA) and diaminobenzidine (Dako A/S, Glostrup, Denmark) as chromogen. Positive and negative controls were included in each slide run. The determination of HER2 overexpression was determined according to GEFPICS guidelines with FISH performed in all cases of HER2 2+ result [38].

### Statistical Analysis

Statistical analyses were performed in both the whole population and in two restricted immune-phenotypic population defined as 1) ERpositive, HER2negative 2) ERpositive, HER2negative, Histological Grade 2.

Time to distant metastases and time to loco-regional recurrences were defined as the time from the breast cancer primary tumour diagnosis to the occurrence of the event. Time to death was defined as the time from the diagnosis of the metastases to the occurrence of the death. Survival analyses were performed using the Kaplan-Meier estimate of the survival function. Comparison between survival curves was performed using the logrank test. Hazard ratios were estimated using the Cox proportional hazard model. P-values were considered significant when below 0.05. Only variables with a significant p-value in univariate analyses were included in a multivariate ascending stepwise procedure using the Cox model.

The multivariate model performance was quantified with respect to discrimination (i.e., whether the relative ranking of individual predictions is in the correct order when compared to observation), quantified with the Concordance index (C-index) [Harrell et al Ref. 1, 1996] and its 95% confidence interval. The analyses were performed using R software (http://cran.r-project.org).

### Ethics Statement

The registration of patients of the Institut Curie (Paris and Saint-Cloud) in this cohort received a favorable agreement of the french National Committee on Computers and Liberties (CNIL, Commission nationale de l'informatique et des libertés). Patients gave informed written consent prior to be registered in the cohort. The study was approved by the breast cancer study group and the comity of clinical research study of the Institut Curie (Paris and Saint-Cloud).

## Results

### Patients

A continuous retrospective series of 456 patients with pN0, pT1–pT2, invasive breast carcinoma, treated at the Institut Curie between 1995–1996 was identified using a prospective database (Table 1). All patients were all treated by lumpectomy plus axillary lymph node dissection. 27 (5.9%) patients received an adjuvant chemotherapy and 32 (8.5%) adjuvant hormonal therapy for 5 years. All of the patients received an irradiation of the whole breast with a median dose of 50 Gy [45–55] (International Comission on Radiation Units; ICRU) in 25 daily fractions and 5 weeks. 347 patients (76%) had a boost to the tumor bed with a median dose of 15 Gy [5]–[27] in 8 daily fractions, and 231 patients (50.6%) received irradiation of the internal mammary chain (combination of photons and electrons) to 45 Gy in 23 daily fractions and 4.6 weeks. The clinical and pathological features of patients are summarized in tables Table 1. Tumors corresponded mainly to ductal (76%) or lobular (14%) infiltrating carcinomas. All cases were free of axillary lymph node metastases. Tumors were classified as histological grade I (HG1) in 35% (161/456), HG2 in 42% (192/456) and HG3 in 23% (103/456) (Notthingham histological grade). Immunophenotyping showed that ER was expressed in 86% (386/456) of the tumors, PR in 70% (319/456), HER2 in 5% (23/456) whereas 14% (62/456) remained negative for all three markers. The median follow-up period was 151 [5–191] months. 73 patients developed a distant relapse (16%) and 81 patients developed a loco-regional recurrence (17.7%). 19 patients (26%) had bone as the only site of metastases when first diagnosed with metastatic disease. Other locations were lung, liver, brain, lymph-node, bowel and skin.

**Table 1:** 456 pN0 breast cancer patients**.**

456 pT1 pT2 pN0 invasive breast cancer patients								
		N (%)	10y LRRFI	p value	RR [95%CI]	10y DDFI	p value	RR [95%CI]
	all	456 (100)	85 [81–88]			85 [82–89]		
Age (years)	>55	219 (48)	89 [84–94]	0.042^*^	1	87 [82–92]	0.27	1
	>40 and ≤55	214 (47)	83 [78–88]		1.3 [0.8–2.1]	85 [80–90]		1.3 [0.8–2.1]
	≤40	23 (5)	66 [49–90]		2.6 [1.2–5.8]	74 [58–94]		1.9 [0.8–4.6]
BMI	BMI 20–25	277 (61)	83 [78–88]	0.5285	1	83 [79–88]	0.06	1
	BMI<20	22 (5)	91 [79–100]		0.8 [0.3–2.3]	95 [85–100]		0.4 [0.1–1.8]
	BMI 25–30	86 (18)	91 [85–98]		0.7 [0.4–1.3]	94 [89–99]		0.5 [0.2–1.07]
	BMI>30	38 (8)	87 [76–100]		0.6 [0.2–1.5]	76 [63–92]		1.6 [0.8–3.2]
Familial	No	376 (83)	86 [82–90]	0.09	1	85 [82–89]	0.84	1
	Yes	79 (17)	80 [71–90]		1.5 [0.9–2.6]	86 [78–94]		1.06 [0.6–1.9]
Menopause	No HRT	195 (43)	89 [85–94]	0.04^*^	1	87 [82–93]	0.26	1
	HRT	80 (17)	84 [76–93]		1.7 [0.9–3.2]	84 [76–93]		1.2 [0.6–2.4]
	Pre-menopausal	181 (40)	81 [75–87]		1.9 [1.2–3.2]	83 [78–89]		1.5 [0.9–2.6]
T	T1	345 (76)	85 [82–89]	0.26	1	86 [83–90]	0.002^*^	1
	T2	111 (24)	82 [74–89]		1.3 [0.8–2.1]	79 [72–88]		2 [1.2–3.2]
pT	pT1	344 (75)	86 [82–90]	0.39	1	86 [82–90]	0.03^*^	1
	pT2	112 (25)	82 [75–90]		1.24 [0.7–2]	81 [74–89]		1.7 [1.1–2.8]
Margin	<3 mm	163 (36)	79 [73–86]	0.007^*^	1	85 [79–91]	0.55	1
	≥3 mm	293 (64)	88 [84–92]		0.5 [0.3–0.8]	85 [81–90]		0.8 [0.5–1.3]
Type	Ductal	346 (76)	85 [81–89]	0.25	1	85 [81–89]	0.03^*^	1
	Lobular	64 (14)	84 [74–94]		1.2 [0.7–2.2]	80 [71–92]		1.3 [0.7–2.3]
	Ductal Lobular	13 (3)	72 [49–100]		1.7 [0.5–5.4]	76 [55–100]		2.2 [0.8–6.2]
	Other	33 (7)	92 [83–100]		0.3 [0.08–1.4]	100 [100–100]		0
LVI	No	385 (84)	86 [82–90]	0.10	1	87 [83–91]	0.01^*^	1
	Yes	71 (16)	80 [71–91]		1.5 [0.9–2.6]	75 [66–87]		1.9 [1.2–3.3]
Mitotic index	I	311	86 [82–90]	0.75	1	90 [86–93]	7e−5	1
	II	58	81 [71–93]		1.2 [0.6–2.2]	75 [64–88]		2.5 [1.4–4.7]
	III	87	84 [76–92]		1.2 [0.7–2]	76 [67–86]		2.7 [1.6–4.6]
EE Grade	I	161 (35)	89 [84–94]	0.11	1	93 [89–97]	2e−4	1
	II	192 (42)	84 [78–90]		1.4 [0.8–2.3]	84 [78–90]		2.4 [1.2–4.6]
	III	103 (23)	80 [72–89]		1.8 [1.04–3.3]	75 [67–84]		4.4 [2.2–8.6]
ER and HER2	ER+ HER2−	371 (81)	86 [82–90]	0.31	1	88 [84–91]	6e−4	1
	HER2+	23 (5)	76 [59–97]		1.6 [0.7–3.7]	61 [44–85]		3.4 [1.7–6.7]
	ER− HER2−	62 (14)	81 [71–92]		1.4 [0.7–2.5]	80 [70–91]		1.6 [0.8–2.9]
Ki67	< = 20	274 (60)	88 [84–92]	0.08	1	92 [88–95]	<1e−6^*^	1
	>20	182 (40)	79 [73–86]		1.5 [0.9–2.3]	74 [68–81]		3 [1.8–4.8]

BMI: body mass index, LN: lymph node, pT: histological size of the invasive carcinoma (pT1≤20 mm, pT2>20vmm), LVI: lympho-vascular involvement, EE: histological grade according to Ellis and Elston, ER: Estradiol Receptor, HER2: Human Epidermal Receptor type 2. LRFI: Loco-Regional Free Interval. DDFI: Distant Disease Free Interval.

ER positive and HER2 negative tumors constituted a subgroup of 371 (81.4%) cases (Table 2). In this group, tumors corresponded mainly to ductal (74%) or lobular (16%) carcinoma. It was classified as HG1 in 41% (153/371), HG2 in 46% (169/371) and HG3 in 13% (49/371) of cases. 50 patients (13.5%) developed a distant relapse and 61 patients (16.4%) developed a loco-regional recurrence. Another subgroup (169 cases, 37%) corresponded to ER positive HER2 negative HG2 tumors (Table 3). In this subgroup, 28 patients (16.5%) developed a distant relapse and 30 (17.7%) a loco-regional recurrence.

**Table 2:** 371 ER positive HER2 negative breast cancer patients.

371 ER positive HER2 negative, pN0 invasive breast cancer patients								
		N (%)	10y LRRFI	p	RR [95%CI]	10y DDFI	p	RR [95%CI]
	all	371	86 [82–90]			88 [84–91]		
Age (years)	>55	190 (51)	90 [85–95]	0.009^*^	1	88 [83–93]	0.69	1
	>40 and ≤55	167 (45)	85 [79–91]		1.2 [0.7–2]	88 [82–93]		1.3 [0.7–2.2]
	≤40	14 (4)	57 [36–90]		3.7 [1.5–9]	85 [68–100]		1.1 [0.2–4.5]
BMI	20–25	233 (63)	83 [78–89]	0.5	1	86 [84–92]	0.03^*^	1
	<20	20 (5)	90 [77–100]		0.9 [0.3–2.7]	94 [80–97]		0.6 [0.1–2.4]
	25–30	67 (18)	95 [90–100]		0.6 [0.3–1.2]	97 [92–100]		0.4 [0.1–1]
	>30	27 (7)	87 [74–100]		0.7 [0.2–2.7]	78 [84–94]		2 [0.9–4.6]
Familial	No	302 (82)	87 [82–91]	0.52	1	88 [81–93]	1	1
	Yes	68 (18)	85 [76–94]		1.2 [0.6–2.2]	88 [90–100]		1 [0.4–2.1]
Menopause	No HRT	171 (46)	91 [86–96]	0.1	1	89 [72–97]	0.4	1
	HRT	66 (18)	85 [76–95]		1.7 [0.8–3.4]	87 [90–98]		1.2 [0.5–2.6]
	Pre-menopausal	134 (36)	81 [75–89]		1.8 [1.04–3.3]	87 [77–89]		1.5 [0.8–2.8]
T	T1	289 (78)	87 [83–92]	0.07	1	89 [86–93]	0.0002^*^	1
	T2	82 (22)	81 [73–91]		1.6 [0.9–2.8]	80 [72–89]		2.8 [1.6–4.9]
pT	pT1	289 (78)	88 [83–92]	0.11	1	89 [85–93]	0.001^*^	1
	pT2	82 (22)	81 [73–91]		1.6 [0.9–2.7]	82 [73–91]		2.5 [1.4–4.4]
Margin	<3 mm	124 (33)	82 [75–89]	0.03^*^	1	87 [80–93]	0.35	1
	≥3 mm	247 (67)	88 [84–93]		0.6 [0.3–0.9]	88 [83–92]		0.7 [0.4–1.3]
Type	Ductal	275 (74)	87 [83–91]	0.26	1	88 [83–92]	0.17	1
	Lobular	60 (16)	84 [75–95]		1.4 [0.7–2.6]	81 [70–92]		1.4 [0.7–2.7]
	Ductal Lobular	11 (3)	69 [45–100]		2.2 [0.7–7.1]	90 [73–100]		1.4 [0.3–7.9]
	Other	25 (7)	90 [78–100]		0.5 [0.1–2.1]	100 [100–100]		0
LVI	No	319 (86)	86 [82–90]	0.59	1	89 [85–92]	0.04^*^	1
	Yes	52 (14)	85 [76–96]		1.2 [0.6–2.3]	79 [68–92]		2 [1.1–3.8]
Mitotic index	I	285 (77)	87 [82–91]	0.93	1	90 [85–94]	0.005^*^	1
	II	48 (13)	82 [71–95]		1.1 [0.5–2.3]	81 [69–93]		3.1 [1.1–4.3]
	III	38 (10)	88 [78–100]		1.1 [0.4–2.5]	83 [71–96]		2.8 [1.4–5.8]
EE Grade	I	153 (41)	88 [83–94]	0.22	1	94 [89–97]	0.003^*^	1
	II	169 (46)	85 [79–91]		1.5 [0.5–4.2]	83 [76–89]		2.5 [1.2–5.1]
	III	49 (13)	83 [72–95]		1.7 [0.6–5.3]	87 [77–97]		3.8 [1.6–8.8]
Ki67	< = 20	248 (67)	88 [83–92]	0.3	1	91 [88–95]	0.0003^*^	1
	>20	63 (45)	82 [75–90]		1.3 [0.8–2.5]	79 [71–87]		2.6 [1.5–4.6]

BMI: body mass index, LN: lymph node, pT: histological size of the invasive carcinoma (pT1≤20 mm, pT2>20 mm), LVI: lympho-vascular involvement, EE: histological grade according to Ellis and Elston, ER: Estradiol Receptor, HER2: Human Epidermal Receptor type 2. LRFI: Loco-Regional Free Interval. DDFI: Distant Disease Free Interval.

**Table 3:** 169 ER positive HER2 negative Histological Grade II, pN0, breast cancer patients.

169 ER positive HER2 negative, Histological Grade II, pN0 invasive breast cancer patients								
		N (%)	10y LRRFI	p	RR [95%CI]	10y DDFI	p	RR [95%CI]
	all	169	85 [79–90]			82 [76–89]		
Age (years)	>55	88 (52)	86 [79–94]	0.2	1	83 [75–91]	0.3	1
	>40 and ≤55	74 (44)	86 [77–94]		1 [0.5–2.2]	80 [70–91]		1.4 [0.7–2.9]
	≤40	7 (4)	57 [30–100]		2.8 [0.8–9.9]	0		0
BMI	20–25	109 (65)	83 [76–91]	0.8	1	79 [72–88]	0.02^*^	1
	<20	8 (5)	73 [47–100]		1.5 [0.3–6.3]	83 [58–100]		0.5 [0.1–4]
	25–30	29 (17)	92 [81–100]		0.8 [0.3–2.2]	0		0
	>30	12 (7)	90 [73–100]		0.5 [0.06–3.5]	70 [46–100]		2 [0.7–6]
Familial	No	140 (83)	84 [78–91]	0.7	1	83 [76–90]	0.4	1
	Yes	28 (16)	87 [74–100]		1.2 [0.5–2.9]	83 [69–99]		1.4 [0.6–3.5]
Menopause	No HRT	76 (45)	87 [79–96]	0.1	1	85 [77–94]	0.3	1
	HRT	34 (20)	90 [80–100]		1.6 [0.6–4.5]	77 [63–93]		2 [0.8–5.4]
	Pre-menopausal	59 (35)	79 [68–90]		2.3 [1–5.2]	82 [72–93]		1.6 [0.6–3.8]
T	T1	127 (75)	84 [77–91]	0.5	1	84 [78–91]	0.04^*^	1
	T2	42 (25)	86 [76–98]		0.7 [0.3–1.8]	76 [64–91]		2 [1–4.5]
pT	pT1	118 (70)	85 [78–92]	0.5	1	84 [77–92]	0.12	1
	pT2	42 (25)	86 [76–98]		0.7 [0.3–1.8]	79 [67–93]		1.8 [0.8–4]
Margin	<3 mm	54 (32)	85 [75–96]	0.5	1	78 [67–92]	0.6	1
	≥3 mm	115 (68)	84 [78–92]		0.7 [0.3–1.6]	84 [77–91]		0.8 [0.4–1.7]
Type	Ductal	120 (71)	85 [79–92]	0.8	1	83 [76–91]	0.5	1
	Lobular	38 (23)	83 [71–98]		1 [0.4–2.5]	75 [61–91]		1.3 [0.6–3]
	Ductal Lobular	9 (5)	73 [47–100]		1.5 [0.3–6.6]	0		0
	Other	2 (1)	0		0	0		0
LVI	No	138 (82)	85 [79–92]	0.4	1	84 [77–90]	0.3	1
	Yes	31 (18)	82 [69–98]		1.4 [0.6–3.3]	76 [60–95]		1.5 [0.6–3.6]
Mitotic index	I	137 (81)	85 [78–91]	0.6	1	85 [79–92]	0.2	1
	II	29 (17)	83 [70–100]		0.9 [0.3–2.3]	73 [58–92]		2 [0.8–4.5]
	III	3 (2)	0		0	66 [30–100]		2.3 [0.3–17]
Ki67	< = 20	102 (60)	85 [78–93]	0.5	1	88 [81–95]	0.07	1
	>20	67 (40)	84 [75–94]		0.8 [0.4–1.7]	75 [65–87]		2 [0.9–4]

BMI: body mass index, LN: lymph node, pT: histological size of the invasive carcinoma (pT1≤20 mm, pT2>20 mm), LVI: lympho-vascular involvement, ER: Estradiol Receptor, HER2: Human Epidermal Receptor type 2. LRFI: Loco-Regional Free Interval. DDFI: Distant Disease Free Interval.

### Histological Grade, Mitotic Index and Ki67 Rate

The kernel density plots of the Mitotic Index (MI) in each grade categories showed a low mitotic index (≤20) for 100% of the HG1 tumors and for 96% of the HG2 tumors. Only HG3 tumors had a Mitotic Index higher than 20 in 80%. Ki67 distribution was a much more discriminatory factor with extreme values in HG1 (90% with Ki67≤20) and HG3 (85% with Ki67>20) tumors. In contrast, a wide spectrum of the Ki67 rate was observed in HG2 tumors: it was ≤20% in 59% of the cases and >20 in 41%. We identified a subgroup of 262 (57%) samples with a low MI (<20) and a low Ki67 rate (≤20), 112 (24%) samples with a low MI and a high Ki67 rate, 77 (15%) samples with high MI and a high Ki67 rate and 12 (3%) samples with high MI and low Ki67 rate.

### Loco Regional Recurrence

**Figure 1:**
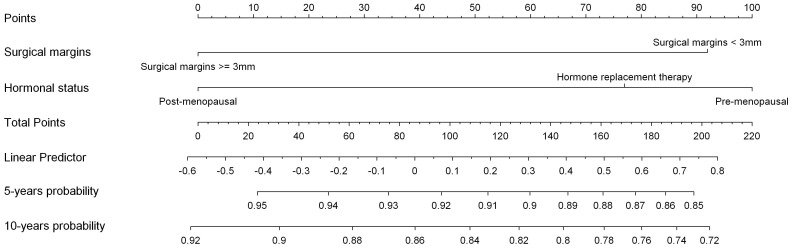
Loco Regional Free Interval Nomogram.

Univariate analyses (Table 1) showed that young age, pre-menopausal status or hormone replacement therapy and non-clear surgical margins (less than 3 mm) were associated with an increased rate of loco-regional recurrences. We performed a subgroup analysis in 371 ER+ HER2− patients (81.4%) and showed that age at diagnosis and surgical margins were still significant factors correlated to an increasing risk of loco-regional recurrence (Table 2). No factors were identified in ER+, HER2−, HG2 tumor samples (Table 3). Variables selected in the multivariate model are summarized in table 4 (in terms of Hazard Ratio, confidence Intervals and p value). Only menopausal status and surgical margins were finally selected (Table 4, figure S1 and S2). The C-index of this model was 0.62 [CI95% = 0.56–0.68]. A nomogram was built (Figure 1).

**Table 4:** Loco Regional Recurrence Free Interval. Multivariate analysis.

Loco Regional Recurrence Free Interval. Multivariate analysis							
		456 pN0			371 ER+ HER2− pN0		
		N (%)	RR	p	N (%)	RR	p
Surgical margin	<3 mm	163 (36)	1	0.006^*^	124 (33)	1	0.048^*^
	≥3 mm	293 (64)	0.5 [0.3–0.8]		247 (67)	0.6 [0.3–0.9]	
Menopause	No HRT	195 (43)	1	0.03^*^			
	HRT	80 (17)	1.6 [0.8–3.1]				
	Pre-menopausal	181 (40)	1.9 [1.2–3.2]				
Age (years)	>55				190 (51)	1	0.06
	>40 & < = 55				167 (45)	1.3 [0.7–2.2]	
	< = 40				14 (4)	3.4 [1.4–8.2]	

### Distant Relapse

Univariate analyses (Table 1) showed that pathological tumor size (p = 0.03), histological type (p = 0.03), lympho-vascular invasion (p = 0.01), histological grade (p = 0.00002), immunophenotypic subtypes (ER+ HER2− and ER−; p = 0.0006), and Ki67 rate (p<10e−6) were associated with an increased rate of distant metastases.

**Figure 2:**
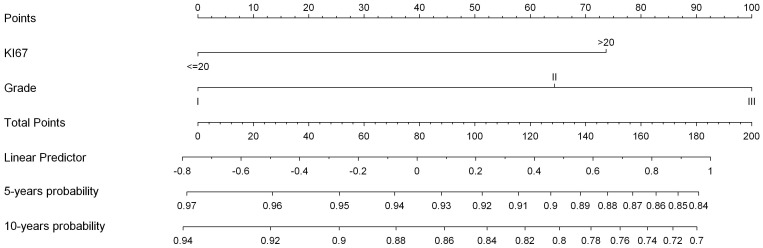
Distant Disease Free Interval Nomogram.

We designed a multivariate ascending stepwise procedure using the Cox model to determine the probability of distant metastasis. In the whole population, Ki67 rate and histological grade remained significant variables (figure S3 and S4). Ki67 rate was the only significant variable when the multivariate analysis was performed in the whole population or in the two subgroups of ER+ HER2− and ER+ HER2− HG2 (Table 2, Table 3, Table 5). C-index of this model was 0.68 [CI95% = 0.62–0.74]. A nomogram was built (Figure 2).

**Table 5:** Distant Metastasis Free interval Multivariate Analysis.

Distant Metastasis Free Interval. Multivariate analysis										
		456 pN0			371 ER+HER2− pN0			169 ER+HER2− HG2 pN0		
		N (%)	RR	p	N (%)	RR	p	N (%)	RR	p
Ki67	< = 20	274 (60)	1	0.01^*^	248 (67)		6e−4	102 (60)	1	0.04^*^
	>20	182 (40)	2 [1.1–3.6]		123 (33)	2.6 [1.5–4.6]		67 (40)	2.2 [1.01–4.8]	
EE	I	161 (35)	1	0.06						
	II	192 (42)	1.8 [0.9–3.7]							
	III	103 (23)	2.6 [1.1–5.8]							

Ki67 rate (%). EE Grade: Histological Grade as defined by Elston Ellis

### From First Metastatic Event to Death

**Figure 3:**
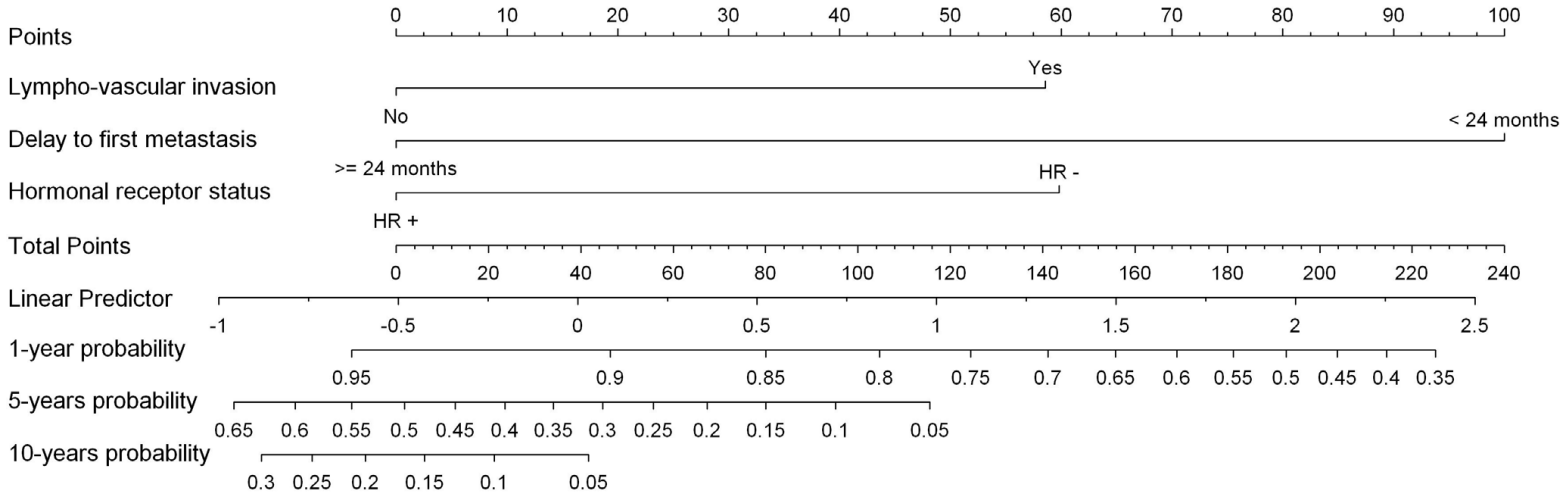
From First Metastatic Event to Death Nomogram.

Development of distant relapse was observed in 73 patients (16%). Median delay from metastasis diagnosis to death was 36 months [1–144]. 19 patients (26%) had metastasis in bone only as first diagnostic of secondary tumor location. Other sites were lung, liver, brain, lymph node, bowel and skin. Primary tumors features (lympho-vascular invasion, histological grade, hormone receptor status), time lapse between primary tumor and first metastasis and first metastasis location (bone versus other locations) were all correlated to the time-lapse from first metastatic event to death from breast cancer (figure S5, S6 and S7). Variables selected in the multivariate model were time-lapse from primary tumor to first metastasis diagnosis, lympho-vascular invasion and hormone receptor status (Table 6). The C-index of the model was 0.66 [CI95% = 0.59–0.74]. A nomogram was built (Figure 3).

**Table 6:** Overall Survival Analysis from First Metastatic Event to Death.

Overall Survival Analysis from Metastasis Event to Death.							
		N (%)	36 months OS	Univariate Analysis		Multivariate Analysis	
				RR	p	RR	p
	All	73	54 [44–68]				
LVI	No	54 (74)	58 [46–73]	1	0.05^*^	1	0.016^*^
	Yes	19 (26)	46 [27–76]	1.7 [1]–[3]		2.1 [1.2–3.8]	
EE Grade	I	12 (16)	82 [63–100]	1	0.03^*^		
	II	32 (44)	58 [42–79]	1.8 [0.8–4.2]			
	III	29 (40)	9 [24–62]	2.8 [1.2–6.3]			
ER	Positive	56 (77)	63 [50–77]	1	0.04^*^		
	Negative	17 (23)	29 [14–61]	1.8 [1–3.4]			
PR	Positive	50 (69)	69 [56–84]	1	0.005^*^		
	Negative	23 (31)	26 [13]–[52]	2.1 [1.2–3.7]			
HR	ER+ or PR+	57 (78)	63 [51–78]	1	0.03^*^	1	0.03^*^
	ER− & PR−	16 (22)	25 [10–58]	2 [1.1–3.7]		2.2 [1.1–4.1]	
ER/HER2	ER+ HER2−	50 (68)	64 [51–80]	1	0.07		
	HER2+	10 (14)	50 [27–93]	1.5 [0.6–3.3]			
	ER− HER2−	13 (18)	23 [8–62]	2 [1.1–4.2]			
Delay (months)	<24	8 (11)	25 [7–83]	1	0.002^*^	1	0.005^*^
	> = 24	65 (89)	58 [47–72]	0.3 [0.1–0.7]		0.3 [0.1–0.6]	
First Metastasis Location	Bone Only	19 (26)	73 [56–93]	1	0.02^*^		
	Other	54 (74)	39 [27–56]	1.8 [1.1–3.2]			

73 metastatic breast cancer patients. LVI: lympho-vascular involvement, EE Grade: Histological Grade as defined by Elston Ellis. ER: Estrogen Receptor, PR: Progesteron Receptor, HER2: Human Epidermal Receptor type 2, Delay: Delay from primary tumour diagnosis to first metastatic event.

## Discussion

This study aimed to analyze the Ki67 rate prognostic value in a large cohort of 456 consecutive early-stage (pT1–pT2), pN0 breast cancer patients. These patients were all treated by primary breast-conserving surgery followed by whole-breast radiotherapy. A few patients received either adjuvant chemotherapy (5.9%) or a 5-year adjuvant hormonal therapy (8.5%). The median follow-up length was 12 years.

### Distant Relapse

In the whole population, the Ki67 rate (threshold 20%) was the most significant factor associated to the distant disease free interval, in univariate and in multivariate analyses, outperforming the values of both Mitotic Index and HG. Ki67 rate was the only significant variable in the subgroups of ER+ HER2− and of ER+ HER2− HG2 tumors. As the concordance between the HG and Ki67 rate was high for HG1 and HG3 tumors and as the prognostic value of the Ki67 was significant in the ER+ HER2− HG2 subgroup (37% of the cases), we conclude that the Ki67 is a cost-effective decision marker for the indication of adjuvant therapy in more than one third of early-stage, pT1–pT2, pN0, breast cancer patients.

Proliferation is a key determinant of both prognosis [2], [11]–[13] and response to adjuvant systemic treatments whether on chemotherapy [37] or aromatase inhibitors [50]. In a series of 2847 HR+ breast cancer patients, Cheang et al showed how Ki67 was able to discriminate luminal B from luminal A tumors and that this marker was significantly associated with poor disease recurrence-free and disease-specific survival in all adjuvant systemic treatment categories [9]. However the determination of the Ki67 threshold remains controversial, ranging from 3 to 35% [13]. The one used in our previous analysis was 20%. The integration of gene expression arrays data and Ki67 immuno-staining allowed us to identify that Ki67 rate higher than 20% was correlated to a strong activation (over-expression) of the genes involved in the tumor proliferation process. It is however still crucial to underline the absolute need to set-up a multi-center, international, standardization process of the determination of Ki67 status. In their letter, Colozza et al [12] expressed their concern that setting Ki67 cut-offs in order to determine the systemic adjuvant therapy, as the St Gallen experts had done at the 2009 Consensus (<15%, 16–30%, >30%) was a little hasty as long as a standardization of the Ki67 status, and particularly of the pre-analytical handling of the tumors was not done [20].

Nottingham histological grade (HG) was the second independent prognostic factor for distant metastases in the whole subpopulation but this marker did not reach statistical significance in the subpopulation of luminal cancers. This confirms data showing that HG is a valuable prognostic factor [22], [32], [51], particularly in early breast cancer without lymph node involvement [10].

We built a nomogram based on Ki67 rate and HG to determine the 5 and 10 years probability of distant metastasis event. The maximum distant metastasis free probability [HG1, low Ki67 rate] was 96% and 92% at 5 and 10 years respectively. The minimum distant metastasis free probability was 84% and 70% at 5 and 10 years respectively.

### Loco Regional Recurrence

In the whole series of 456 patients, we showed that young age, pre-menopausal status or hormone replacement therapy and non-clear surgical margins (less than 3 mm) were associated with an increased rate of loco-regional recurrences. Ki67 rate was not a factor associated to the loco-regional recurrence free interval. We built a multivariate model and corresponding nomogram based on menopause status and surgical margins to predict the 5 and 10 years loco-regional recurrence probability. The maximum loco-regional free probability [margin> = 3 mm, post-menopause status] was 95% and 92% at 5 and 10 years respectively. The minimum loco-regional free probability [margin<3 mm, pre-menopause status] was 85% and 72% at 5 and 10 years respectively. Many authors have already reported that young age, defined in either three classes or according to the menopause status, and a satisfactory surgical margin (3 mm) were major prognostic factors associated with loco-regional recurrence [22], [29], [31]. Macroscopic involvement of the margin has been associated, since the eighties, with an increased risk of developing local recurrences despite the use of radiotherapy [18], [30]. The impact of inadequate surgical margins seem to be lessened by postoperative radiotherapy [24] even though it is not eradicated [33], [52]. Fourquet et al [18] showed in a series of 518 patients, of whom 68% were premenopausal, treated by breast conserving surgery followed by whole-breast radiotherapy for breast cancers without clinical axillary lymph node involvement that macroscopic involvement of the margin was, after age, the second most important independent factor for local recurrence. The effect of microscopically involved margin by invasive tumours is, on the other hand, more controversial. Many large series of breast conserving surgery with whole-breast radiotherapy have revealed that it was significantly associated, in univariate or multivariate analyses, with a higher rate of local relapses [19], [22], [24]–[27], [29], [31], [33], [36], [42], [43], [52]. Two retrospective studies, performed at the MD Anderson Cancer Center and at the Institut Curie Cancer Center showed that young age remains a major prognostic factor of local recurrence in a population of patients younger than 40 treated by breast conserving surgery and radiotherapy performed as either their initial treatments [7], or after neoadjuvant chemotherapy [34]. The fact that young age is the most significant prognostic factor is not yet understood despite numerous studies. We could find its explanation in tumor biology and/or the hormonal environment specific to pre-menopausal women [1], [6], [28]. The fact that we identified menopausal patients receiving Hormone Replacement Therapy as at the same risk of loco-regional recurrence as pre-menopausal patients seems to strengthen the hormonal environment hypothesis.

### From First Metastatic Event to Death

Finally, we identified that hormonal receptor status, lympho vascular invasion, bone metastasis and the late discovery of the first metastases were significant variables correlated to the time lapse from a first metastatic event to death from breast cancer. We built a multivariate Cox model and corresponding nomogram based on time-lapse from primary tumour to first metastatic diagnosis, and two primary tumor features (lympho vascular invasion and hormone receptor status) to predict the 1, 5 and 10 years probabilities of death from breast cancer. The minimum death probability [time-lapse >24 months, no lympho vascular invasion, hormone receptor positive status] was 5%, 50% and 85% at 1, 5 and 10 years respectively. The maximum death probability [time-lapse <24 months, lympho vascular invasion, hormone receptor negative status] was 70% and 100% at 1 and 5 years respectively. Several groups have previously identified these factors. Chang et al [8], Rowlings et al [41], Rizzieri et al [40], Hortobagyi et al [23] showed that a short disease free interval, ER, PR and HER2 status were correlated to the survival outcome.

## Conclusions

In conclusion, our study confirms the validity of the Ki67 proliferation marker to better evaluate the risk of distant metastases in early stage, pT1–pT2, pN0 breast cancers. Ki67 was not a relevant prognostic factor of loco-regional recurrence or of the time-lapse between the diagnosis of first metastasis and death. Since the concordance between the HG and Ki67 rate was high for HG1 and HG3 tumors and since the prognostic value regarding distant relapse of Ki67 rate was significant in the ER+ HER2− HG2 subgroup, we concluded that the Ki67 rate is a potential cost-effective prognostic proliferation marker in this later subgroup which represents 37% of early stage pN0 breast cancer patients. Three nomograms were built from this study to determine the probability of metastatic relapse, loco-regional recurrence and death from breast cancer at the time of first metastases diagnosis.

## Supporting Information

Figure S1 **Loco Regional Free Interval.** Kaplan Meier Curves. Menopausal status.
(PDF)

Figure S2 **Loco Regional Free Interval.** Kaplan Meier Curves. Surgical Margin.
(PDF)

Figure S3 **Distant Disease Free Interval.** Kaplan Meier Curves. Ki67 rate.
(PDF)

Figure S4 **Distant Disease Free Interval.** Kaplan Meier Curves. Histological Grade
(PDF)

Figure S5 **First Metastatic Event to Death.** Kaplan Meier Curves. Hormone Receptors
(PDF)

Figure S6 **First Metastatic Event to Death.** Kaplan Meier Curves. Lympho-Vascular Invasion
(PDF)

Figure S7 **First Metastatic Event to Death.** Kaplan Meier Curves. Delay.
(PDF)
