# Supplementary figures and images for: Long-Term Prognostic Performance of Ki67 Rate in Early Stage, pT1-pT2, pN0, Invasive Breast Carcinoma
Source: PLoS One. 2013 Mar 19;8(3):e55901. doi: 10.1371/journal.pone.0055901 (PMC3602517; doi:10.1371/journal.pone.0055901)

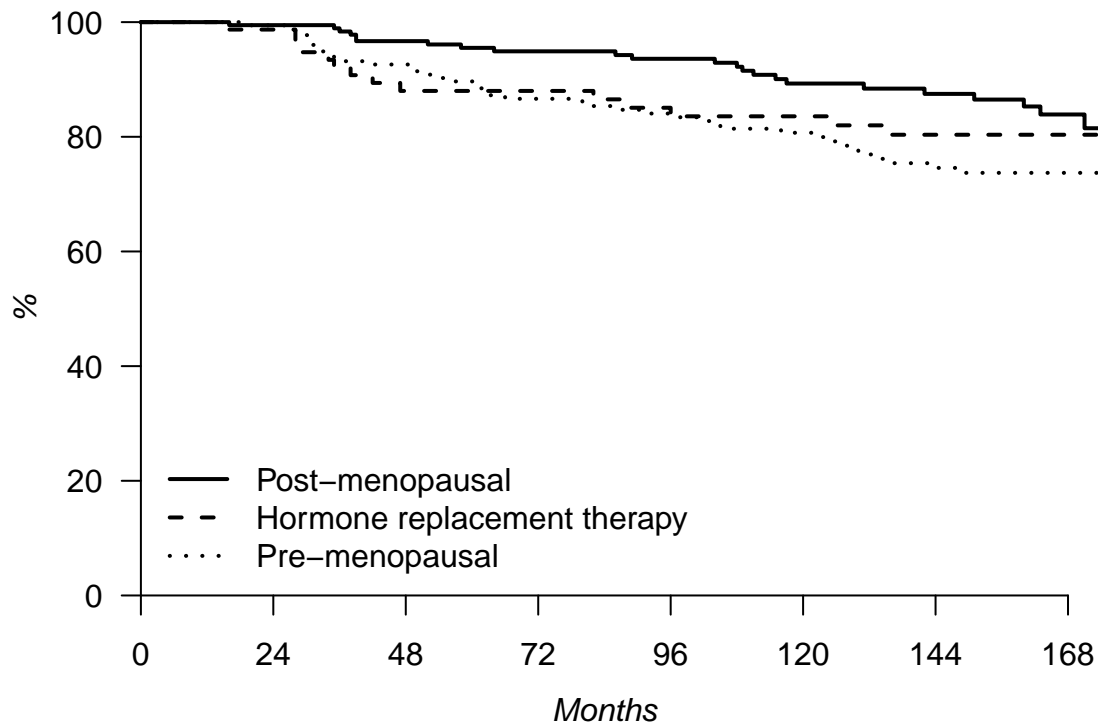

### Number at risk

|     |     |     |     |     |     |     |    |    |
|-----|-----|-----|-----|-----|-----|-----|----|----|
| —   | 195 | 186 | 166 | 154 | 141 | 113 | 94 | 45 |
| - - | 80  | 75  | 63  | 61  | 57  | 54  | 42 | 20 |
| ... | 181 | 176 | 158 | 138 | 129 | 110 | 92 | 52 |

Supplement: Figure S1 — Loco Regional Free Interval. Kaplan Meier Curves. Menopausal status. (PDF) [file pone.0055901.s001.pdf]

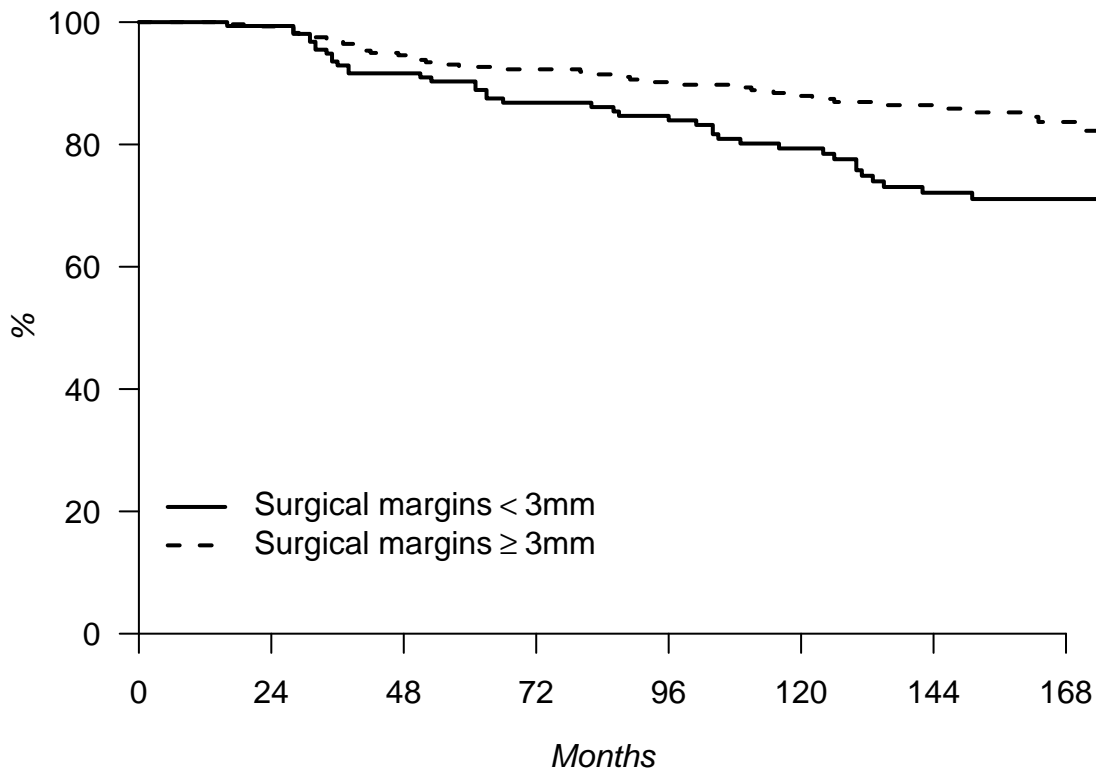

**Number at risk**

|     |     |     |     |     |     |     |     |    |
|-----|-----|-----|-----|-----|-----|-----|-----|----|
| —   | 163 | 155 | 138 | 123 | 115 | 96  | 77  | 41 |
| - - | 293 | 282 | 249 | 230 | 212 | 181 | 151 | 76 |

Supplement: Figure S2 — Loco Regional Free Interval. Kaplan Meier Curves. Surgical Margin. (PDF) [file pone.0055901.s002.pdf]

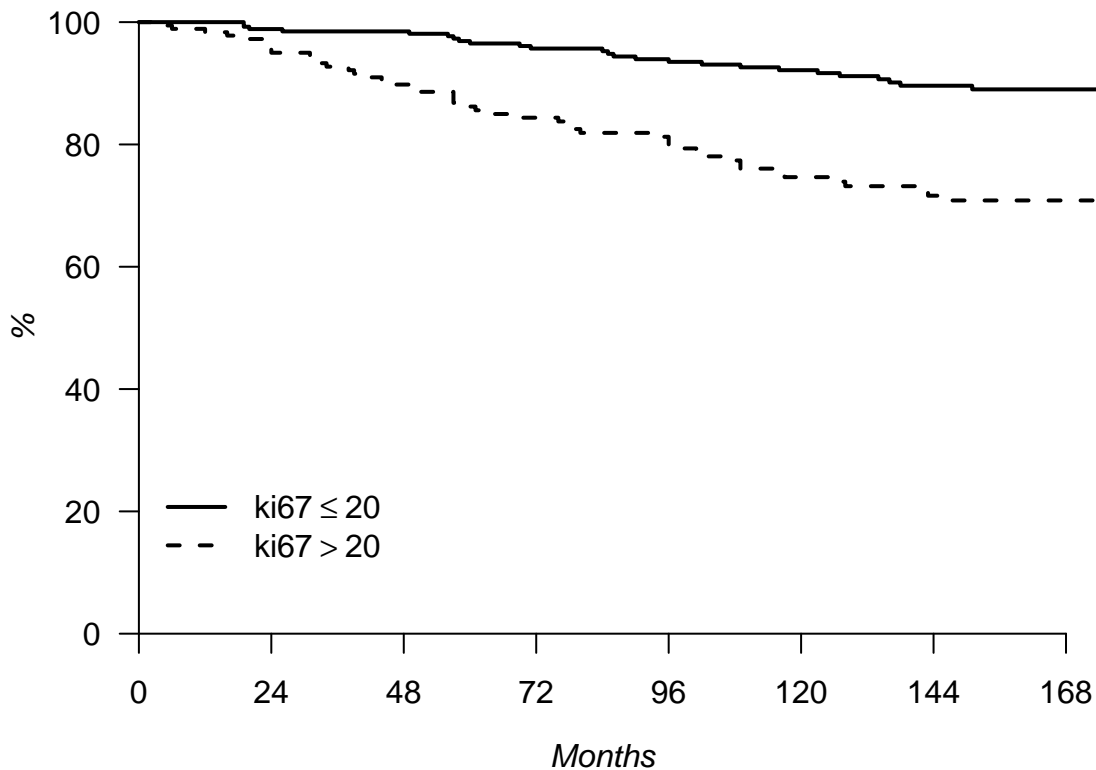

**Number at risk**

|     |     |     |     |     |     |     |     |    |
|-----|-----|-----|-----|-----|-----|-----|-----|----|
| —   | 274 | 260 | 251 | 230 | 215 | 194 | 163 | 83 |
| - - | 182 | 173 | 151 | 137 | 128 | 105 | 92  | 58 |

Supplement: Figure S3 — Distant Disease Free Interval. Kaplan Meier Curves. Ki67 rate. (PDF) [file pone.0055901.s003.pdf]

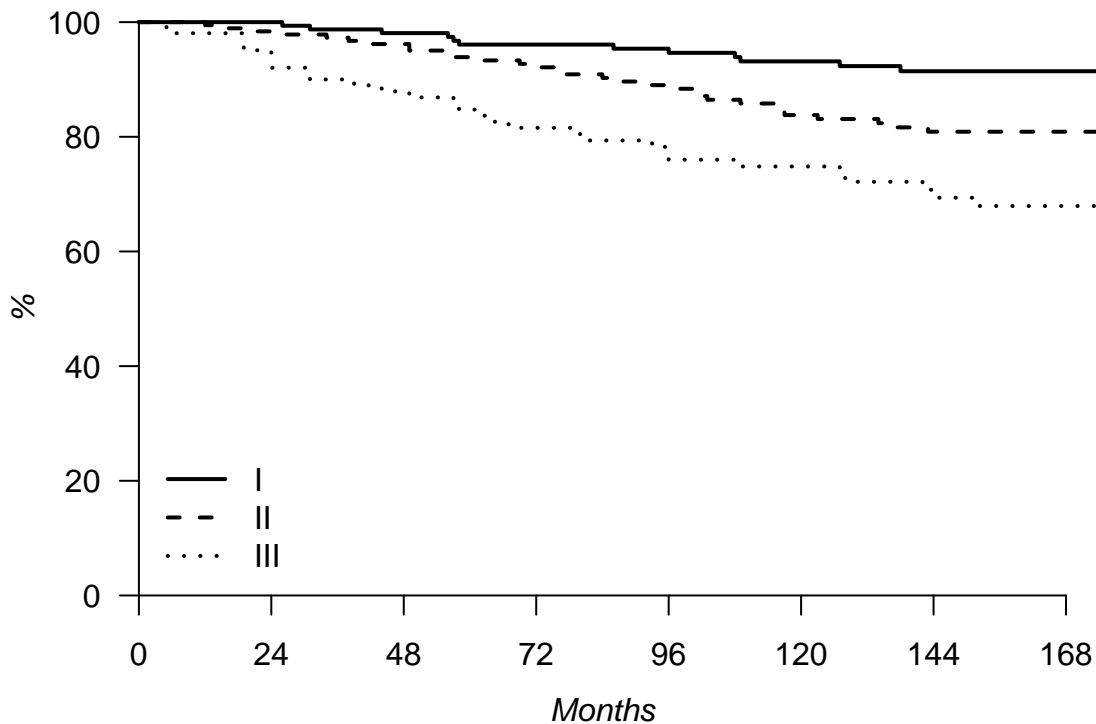

### Number at risk

|     |     |     |     |     |     |     |     |    |
|-----|-----|-----|-----|-----|-----|-----|-----|----|
| —   | 161 | 159 | 150 | 138 | 132 | 118 | 98  | 53 |
| - - | 192 | 180 | 169 | 154 | 141 | 123 | 106 | 58 |
| ... | 103 | 94  | 83  | 75  | 70  | 58  | 51  | 30 |

Supplement: Figure S4 — Distant Disease Free Interval. Kaplan Meier Curves. Histological Grade (PDF) [file pone.0055901.s004.pdf]

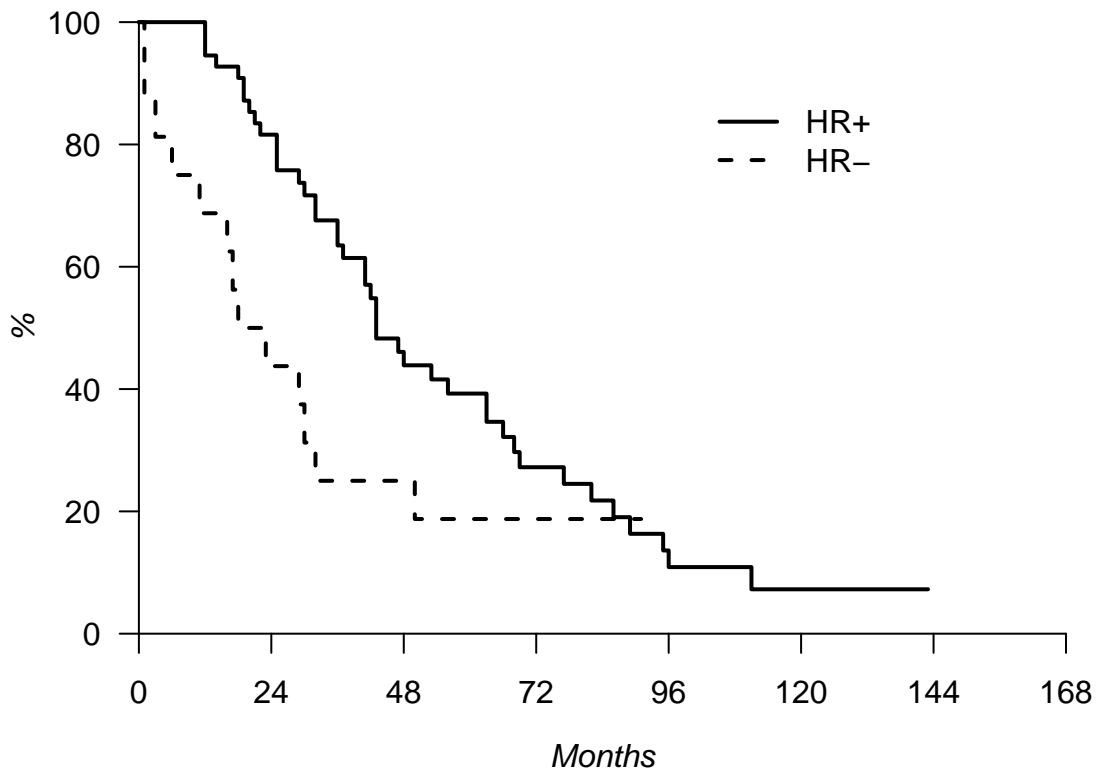

**Number at risk**

|     |    |    |    |    |   |   |   |   |
|-----|----|----|----|----|---|---|---|---|
| —   | 57 | 44 | 21 | 11 | 5 | 2 | 0 | 0 |
| - - | 16 | 7  | 4  | 2  | 0 | 0 | 0 | 0 |

Supplement: Figure S5 — First Metastatic Event to Death. Kaplan Meier Curves. Hormone Receptors (PDF) [file pone.0055901.s005.pdf]

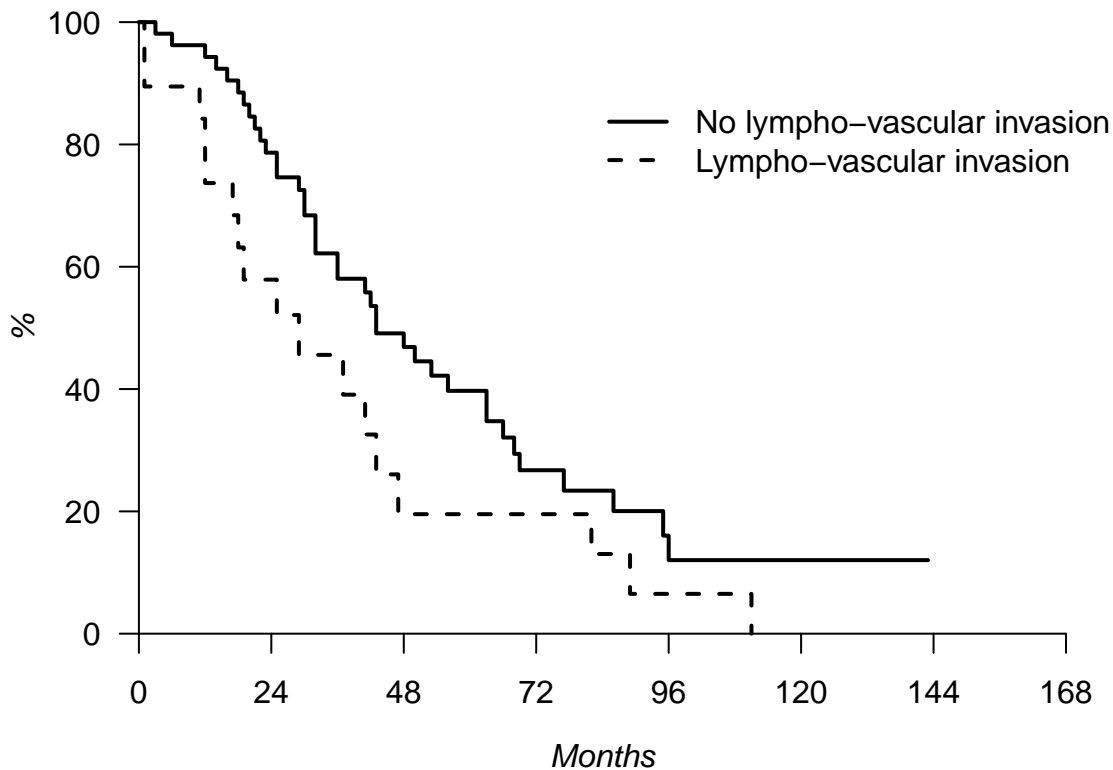

**Number at risk**

|     |    |    |    |    |   |   |   |   |
|-----|----|----|----|----|---|---|---|---|
| —   | 54 | 40 | 22 | 10 | 4 | 2 | 0 | 0 |
| - - | 19 | 11 | 3  | 3  | 1 | 0 | 0 | 0 |

Supplement: Figure S6 — First Metastatic Event to Death. Kaplan Meier Curves. Lympho-Vascular Invasion (PDF) [file pone.0055901.s006.pdf]

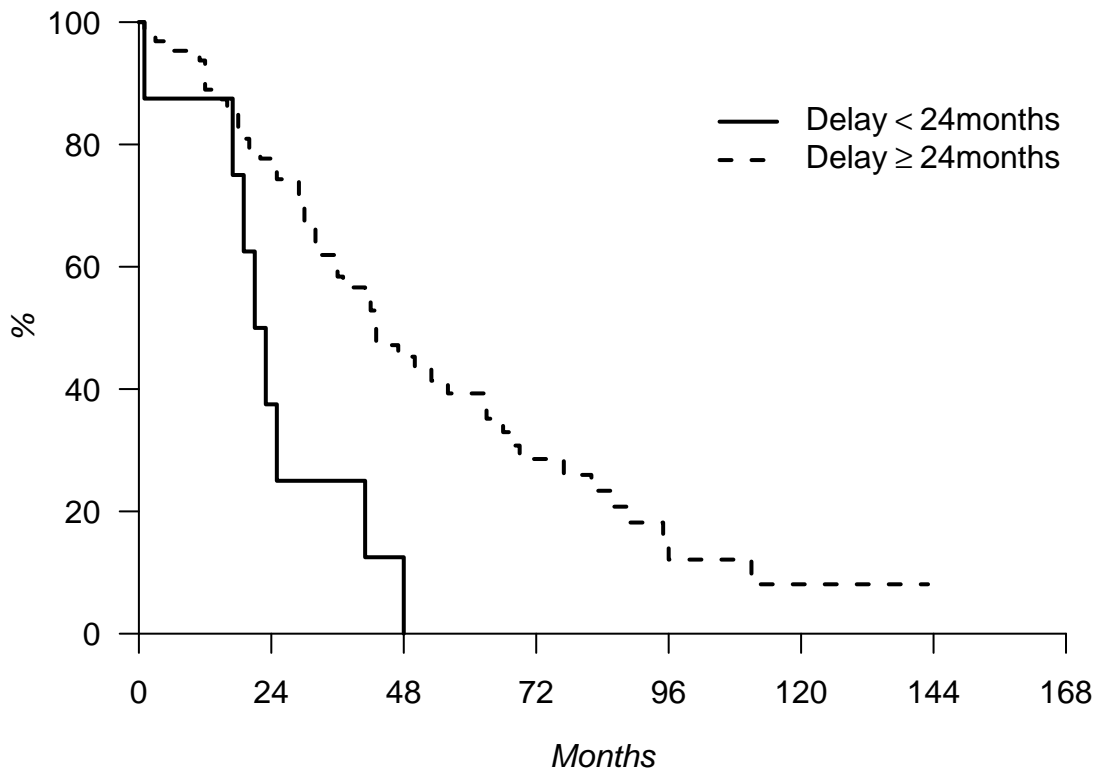

### Number at risk

|     |    |    |    |    |   |   |   |   |
|-----|----|----|----|----|---|---|---|---|
| —   | 8  | 3  | 1  | 0  | 0 | 0 | 0 | 0 |
| - - | 65 | 48 | 24 | 13 | 5 | 2 | 0 | 0 |

Supplement: Figure S7 — First Metastatic Event to Death. Kaplan Meier Curves. Delay. (PDF) [file pone.0055901.s007.pdf]
